# Supplementary material for: Social Disruption Impairs Predatory Threat Assessment in African Elephants
Source: Animals (Basel). 2022 Feb 17;12(4):495. doi: 10.3390/ani12040495 (PMC8868390; doi:10.3390/ani12040495)
Supplement: Supplementary file 1 [file animals-12-00495-s001.zip › animals-1423868-supplementary.pdf]

Supplementary information: Social disruption impairs predatory threat assessment in African elephants

Graeme Shannon[1*], Line S. Cordes[2], Rob Slotow[3] and Cynthia Moss[4] & Karen McComb[5*]

Table S1. Group size, composition and age of matriarch of the elephant families in Pilanesberg (PNP) and Amboseli (ANP), as well as the number of playback experiments conducted.

Population	Elephant group	Group size	Age matriarch*	No. adult females	No. of playbacks
PNP	Abby	4	32	1	3
PNP	Bite	6	26	2	2
PNP	Black	6	32	2	4
PNP	Blue	7	30	2	4
PNP	Gold	11	31	6	3
PNP	Green	11	35	3	4
PNP	Green yellow	10	35	3	6
PNP	V	7	27	2	1
PNP	Monica	4	27	2	1
PNP	Nose	6	27	2	3
PNP	Olivia	15	24	4	4
PNP	Silver	14	33	4	3
PNP	Spear	4	32	1	5
PNP	White	15	33	5	6
PNP	Yellow	7	47	3	4
ANP	AC	8	40	3	2
ANP	BB2	8	37	2	3
ANP	BC	20	41	6	1
ANP	CB	15	46	4	2
ANP	CB2	9	35	3	3
ANP	GB	14	46	5	1
ANP	HA	4	37	2	1
ANP	HB	17	47	5	1
ANP	IAIC	8	47	4	2
ANP	JB	13	34	4	1
ANP	KA	14	38	5	1
ANP	KB3	9	24	3	2
ANP	LA	11	27	4	2
ANP	LB	15	44	5	1
ANP	LC	14	42	5	2

ANP	LD	16	41	5	2
ANP	OA2	5	40	2	2
ANP	PA3	7	29	3	1
ANP	PC2	5	26	2	1
ANP	RA2	9	39	2	1
ANP	SA2	5	34	2	1
ANP	TA	10	40	3	2
ANP	UA2	8	44	3	2
ANP	VA	18	43	6	2
ANP	XA	8	41	3	1

*Demographic data is from 2010 at the end of the study.

Table S2. The observed relationship between each response variable and the model-averaged parameters from the top models (**β**-estimate ± 95% CI)

Response	Pop	β_{3_lions}	β_{n_adults}	β_{age_mat}
**Bunching**	ANP	**0.40 (0.09/0.70)**		
	PNP	0.16 (-0.08/0.39)	**0.11 (0.04/0.19)**	
**Prolonged listening**	ANP	0.24 (-0.09/0.56)	**0.08 (0.01/0.14)**	
	PNP	0.17 (-0.12/0.46)		
**Bunching intensity**	ANP	**2.37 (0.33/4.41)**	0.47 (-0.16/1.11)	0.10 (-0.10/0.29)
	PNP	0.84 (-0.46/2.14)	0.42 (0.09/0.75)	0.00 (-0.12, 0.13)

Bold text denotes β-estimates with 95% CI that do not overlap zero.
